# Comparison of the Degree of Hydrolytic and Cytolytic Modification in Wheat Malts Obtained from Grain of Selected Wheat Cultivars Produced at Different Levels of Nitrogen Fertilisation

**DOI:** 10.3390/molecules30091921

**Published:** 2025-04-25

**Authors:** Józef Gorzelany, Justyna Belcar

**Affiliations:** Department of Food and Agriculture Production Engineering, University of Rzeszów, St. Zelwerowicza 4, 35-601 Rzeszów, Poland; jgorzelany@ur.edu.pl

**Keywords:** wheat grain, malting, extract content, diastatic power, wort attenuation, wort viscosity, nitrogen fertilisation

## Abstract

The degree of hydrolytic modification (marked by parameters: extractive capacity, diastatic power and degree of fermentation) in wheat malts significantly influences their quality, determining their potential for use in brewing. Nitrogen fertilisation at a dose of 60 kg N·ha^−1^ applied in 3-year field experiments had a positive effect on the extractability value of wheat malts, with an average value of 84.51% d.m. The value of diastatic power in the obtained malts, depending on the variety, was on average at the level of 334–414 units W-K (Windisch–Kolbach) and 357–380 units W-K, depending on nitrogen fertilisation. Wort attenuation obtained from the analysed wheat malts was at a similar level (on average 78.1%), except for malt obtained from grain of wheat fertilised with a nitrogen dose of 40 kg N·ha^−1^, for which significantly lower values were obtained (respectively, by 5.12%). The viscosity parameter of wheat malts, determining the degree of cytolytic modification, varied and averaged 1.95 mPa·s for the variety and for the nitrogen fertilisation applied. In 2-year canopy experiments, at a nitrogen fertilisation level of 60 kg N·ha^−1^, the Elixer cultivar was characterised by the best indicators of the degree of hydrolytic and cytolytic modification.

## 1. Introduction

Fertilisation is one of the basic activities performed during the cereal growing season [1,2,3]. The availability of assimilable forms of nutrients regardless of the agricultural production system is one of the most important factors influencing the yield and quality. Wheat cultivation requires the soil to contain at least an average availability of the minerals phosphorus, potassium and magnesium. Wheat fertilisation can be applied in two ways: in solid form and in liquid form applied foliarly or by topdressing [3]. Nowadays, more and more farms, especially large-scale farms, apply fertilisers in liquid form due to, among other things, better assimilability by plants, precise application of fertilisers adapted to the nutritional needs of the plant or lower production cost of fertilisers in liquid form compared to the production of solid fertilisers [1,3,4].

The following parameters are used to determine the degree of hydrolytic (amylolytic) modification: extractivity, diastatic power and degree of fermentation. Important from the point of view of the suitability of wheat for malting purposes is the starch content of the grain, which is correlated with the proportion of protein. The starch fraction is largely responsible for the attenuation of the wort and for obtaining a high extract yield [5]. During the malting process, amylolytic enzymes break down the starch into fermentable sugars that provide the raw material for the yeast in alcoholic fermentation [6]. Wheat malts should use wheat varieties that have a high starch content in dry matter [7]. The ratio of amylopectin to amylose in wheat starch grains is very important for malting and brewing wheat beer. Jin et al. [7] observed that wheat grains with high starch content and amylopectin/amylose ratio averaging 3.75:1 and low total protein content were characterised by very high malting quality. The main enzymes responsible for the biochemical transformations occurring during germination are α-amylase, β-amylase, β-glucanase or proteases. Starch consists of two fractions: linear amylose and branched amylopectin. During germination of cereal grains, α-amylase synthesises mainly in the aleurone layer and hydrolyses the random α-1,4-d-glycosidic bonds found inside amylose into dextrins consisting of six glucose monomers. It also cleaves α-1,4-d-glycosidic bonds found inside amylopectin (starch liquefaction). The α-amylase enzyme is activated during wheat grain germination in aleurone cells. The conditions of the malting process (time and temperature of soaking and germination of the grain, or oxygen content) significantly affect the content and activity of this enzyme in the malted grain. β-amylase is an enzyme contained in the endosperm of wheat grain. It has its highest activity on days 2 and 3 of germination, where it hydrolyses every other α-1,4-d- glycosidic bond, causing the maltose molecule to dissociate from the non-reducing ends of the amylose chain [7,8]. Both α-amylase and β-amylase are unable to cleave the α-1,6-d- glycosidic bond found in branched amylopectin, whereas endoamylase has the ability to bypass the non-hydrolysable bond found in amylopectin and, by hitting the α-1 bond, 4 leads to the formation of a border dextrin in which the non-reducing ends are hydrolysed by exoamylase to maltose, isomaltose and a small amount of glucose [5]. The aim of this study was to determine the degree of amylolytic modification of malts obtained from the grain of different winter wheat cultivars grown under different variants of nitrogen fertilisation.

Through the malt extractability parameter, the amount of substances contained in the malt that pass into the wort during mashing is determined. The extract obtained depends on the enzymatic activity and the chemical composition of the grain [9]. On the basis of the value obtained, brewers select the process parameters and the amount of raw material charge, in order to obtain an appropriate brewhouse yield. Malts characterised by a low extract value are undesirable in brewing, as they increase the raw material mass needed to produce a certain volume of beer, which is associated with an increase in the cost intensity of production [6]. The higher the malt extractivity, the fuller the flavour of the resulting beer, while a high proportion of protein in malt lowers the extract yield. The extract yield depends on the starch content of the malted grain and its structure (amylose/amylopectin ratio), as well as the amount of fermentable sugars formed (starch hydrolysis products; Jin et al. [7]). Good quality wheat malts should have an extractability of more than 83% d.m. [5].

The diastatic power parameter of wheat malt determines the total activity of enzymes from the group of hydrolases that break down starch to glucose oligomers, which serve as the metabolic substrate of beer yeast. It is expressed in Windisch–Kolbach units (W-K). The higher the diastatic power value (minimum value of 250 W-K), the better the enzymatic potential of the malt and the faster the starch hydrolysis process. Malts characterised by a low value of this parameter contain a lot of unhydrolysed starch and the beer obtained from them will have a low degree of final fermentation [9].

Based on the value of the degree of final attenuation of the laboratory wort derived from wheat malt, it is possible to determine the quality of the fermentation process affecting the yield and alcohol yield of a batch of raw material. The variation in the attenuation degree values is related both to the selection of the wheat variety from which the malt was obtained and the malting time of the grain, the malting technique and, to a lesser extent, to other factors [10]. For wheat malts, the degree of attenuation should be characterised by a minimum value of 79% [5].

Contained mainly in the cell walls of cereal grains are non-starchy polysaccharides belonging to the pentosan group: glucans are hydrolysed by β-glucanase, while arabinoxylans are hydrolysed by arabinosidase, xylanosidase, β-d-xylanopyranosidase in combination with acetic and ferulic acid esterases and α-l-arabinofuranosidase, which are synthesised during grain germination [5,8,11,12]. The occurrence of arabinoxylans characterised by high molecular weight in beer wort increases the viscosity and filtration difficulty of the solution and may lead to earlier yeast flocculation, but at the same time water-soluble arabinoxylans influence the sensory profile of the resulting beer and stabilise the beer foam [8,11,13]. Wheat grain contains about 6.6% arabinoxylans (1.5–2.5% in endosperm) and about 0.31–6.70% β-glucans (depending on the wheat variety; [5,9]). The high proportion of arabinoxylans (mainly arabinose and xylose in a ratio of 0.5–0.6:1 in wheat endosperm) in wort and wheat beer has a positive effect on the sensory profile of the final product and also influences beer haze and beer foam formation. Arabinoxylans in beer can be used as a prebiotic in inflammatory bowel disease or as a serum glucose-lowering agent [5,11].

The activity of cytolytic enzymes, as well as the presence of non-starchy polysaccharides (mainly arabinoxylans and, to a lesser extent, β-glucans) and their soluble forms in laboratory wort obtained from wheat malts affect the viscosity parameter. Both run-off time and filtration rate can be determined from the value of the parameter [5,9]. A high value of the viscosity parameter negatively affects the fermentation process and can increase the proportion of sludge in the stored beer. Laboratory wort viscosity for wheat malt should not exceed 1.80 mPa∙s [5]. The aim of this study was to determine the degree of cytolytic modification of malts obtained from the grain of different winter wheat cultivars grown under different variants of nitrogen fertilisation.

## 2. Results

### 2.1. Extract Content in Wheat Malts

The average extractable value of wheat malts during the study years was 82.41% d.m. (Table 1). The extract content of wheat malts depended on the cultivar used and the level of nitrogen fertilisation and was dependent on the growing season. Malt obtained from winter wheat grain of Elixer cultivar was characterised by the lowest average extract content (78.84% d.m.), while for the other analysed wheat malts, the values of the analysed parameter were in the range from 83.39 to 84.06% d.m. A significant effect of the applied nitrogen fertilisation at the dose of 60 kg N·ha^−1^ (variant II) on the extract content of the analysed wheat malts in relation to the control (increase by 5.73% on average) was recorded, while for the other fertilisation variants from the experimental objects, this relation was not significantly differentiated. When analysing the extractivity of wheat malts in individual growing seasons, it was significantly differentiated only for the third year of the study, for which the highest value was obtained (85.20% d.m.) and was on average 6.30% higher than the average value obtained in the second growing season (79.83% d.m.).

### 2.2. Diastatic Power in Wheat Malts

Based on the field tests carried out in 2020–2023, the average value of diastatic power in the analysed wheat malts was 369 units W-K (Table 2). The value of the diastatic power of wheat malt depended on the level of nitrogen fertilisation, the variety used and the growing season. Malt obtained from the winter wheat grain of the Gimantis cultivar was characterised by the lowest value of diastatic power (334 units W-K), while the significantly highest average value was characterised by malt obtained from wheat grain of the Rockefeller cultivar (414 units W-K); an increase of 19.32%. There was a significant effect of applied nitrogen fertilisation at a dose of 60 kg N·ha^−1^ (variant II) and 80 kg N·ha^−1^ (variant III) on the value of diastatic power in the analysed wheat malts compared to the control (increase by 5.56% and 6.05%, respectively). The value of diastatic power in wheat malts obtained from the grain of the analysed winter wheat cultivars was significantly differentiated in the individual growing seasons and amounted, respectively, in the first year of the study to 423 units W-K, in the second year to 373 units W-K and in the third year to 312 units W-K.

### 2.3. Degree of Final Attenuation of Wheat Malts

The average value of the degree of attenuation in the years of the study was 77.1% (Table 3). The value of the degree of attenuation in worts obtained from the analysed wheat malts depended on the cultivar and the applied nitrogen fertilisation (variant I) and was dependent on the growing season. Wort obtained from malt obtained from grain of winter wheat cv. Lawina was characterised by the lowest average attenuation (74.2%), while among the analysed wheat worts, malt obtained from grain of wheat cv. Gimantis was characterised by the significantly highest attenuation value (79.8%); the average attenuation of wort increased by 7.02% in relation to the other obtained worts. The applied nitrogen fertilisation at a dose of 40 kg N·ha^−1^ significantly affected the value of the degree of attenuation in relation to the control (increase in attenuation by 5.12%), while for the other variants of nitrogen fertilisation, this was not a significantly differentiated relationship. The degree of wort attenuation varied between growing seasons, but significantly, the lowest attenuation value was recorded for the first growing season compared to the following two years of the study.

### 2.4. Viscosity of Worts

Based on the results obtained, the average viscosity value of wheat worts in the years of the study was 1.94 mPa·s (Table 4). The value of viscosity in worts obtained from the analysed wheat malts depended on the variety and the level of nitrogen fertilisation, while it did not depend on the growing season. Wort obtained from malt obtained from grain of winter wheat cv. Rockefeller was characterised by the lowest average viscosity (1.85 mPa·s), while among the analysed wheat worts, malts obtained from grain of wheat cv. Gimantis (2.02 mPa·s) and malt from grain of wheat cv. Lawina (2.01 mPa·s) were significantly characterised by the highest viscosity value. The differentiated doses of nitrogen fertilisation applied significantly increased the viscosity values in the analysed wheat malts compared to the control; on average by 5.18% (variant I: dose of 40 kg N·ha^−1^), by 10.73% (variant II: dose of 60 kg N·ha^−1^) and by 7.11% (variant III: dose of 80 kg N·ha^−1^). In addition, significantly different average viscosity values of wheat worts were recorded for variants I and II of nitrogen fertilisation; wort viscosity values were 1.93 mPa·s and 2.05 mPa·s, respectively.

### 2.5. Amylolytic Modification in Wheat Malts from Commodity Fields Experiment

The average extractability of wheat malt obtained from wheat grain of the Elixer cultivar was 78.88% d.m., while its lowest value was obtained in the second year of the study from canopy experiments on farm B and D (75.49–75.50% d.m.). The diastatic power content averaged 365 units W-K, with the lowest value obtained in the first year on farm G and in the second year on farm B. The average attenuation of worts produced from wheat malt obtained from winter wheat grain of the Elixer cultivar during the years of the study was 78.1%, and the highest value was obtained for wheat malt produced from winter wheat grain in the second year of the study in farm B. Comparing the average extractivity, diastatic power and attenuation of wheat malts obtained from winter wheat grain of the Elixer cv. from field and canopy experiments, it was observed that the extractivity of wheat malts from canopy experiments was at a similar level, diastatic power was 6.17% lower and attenuation was 2.41% higher (Table 1, Table 2, Table 3, Table 4 and Table 5).

The average extractability of wheat malt obtained from wheat grain of the Lawina cultivar was 82.09% d.m., while its lowest value was obtained in the first year of the canopy study in farm F (77.33% d.m.). The average value of diastatic power was 314 units W-K, while the lowest value was obtained in the first year of the study in farm C (301 units W-K). The average attenuation of worts produced from wheat malt obtained from winter wheat grain of the Lawina cultivar during the years of the study was 75.5%, and the highest value was obtained for wheat malt produced from winter wheat grain in the first year of the study in farm E. Comparing the average extractivity, diastatic power and attenuation of wheat malts obtained from winter wheat grain of the Lawina cv. from field experiments and from canopy experiments, it was observed that extractivity of wheat malt from canopy experiments was at a similar level, diastatic power was 7.37% lower and attenuation was at a similar level (Table 1, Table 2, Table 3, Table 4 and Table 5).

The average extractability of wheat malt obtained from wheat grain of the Gimantis cultivar was 82.91% d.m., while the lowest value was obtained in the second year of the field tests on farm E (78.75% d.m.). The average value of diastatic power was 302 W-K units. The average attenuation of worts produced from wheat malt of the Gimantis cultivar of winter wheat in the years of the study was 76.1%, and the highest value was obtained for wheat malt produced from winter wheat in the second year of the study on farm E. Comparing the extractivity, diastatic power and attenuation of wheat malts obtained from the Gimantis cultivar of winter wheat from field and canopy experiments, it was observed that the average extractivity of wheat malt from canopy experiments was at a similar level, while the average diastatic power and attenuation were lower by 9.58% and 4.64%, respectively, in relation to the discussed parameter from field experiments in the analysed years of the study (Table 1, Table 2, Table 3, Table 4 and Table 5).

The average extractivity of wheat malt obtained from Rockefeller wheat grain was 82.08% d.m., while the lowest value was obtained in the second year of research on farm C (73.34% d.m.). The average value of diastatic power was 379 units W-K, and the lowest value was obtained during the two years of research on farm E. The average attenuation of worts produced from wheat malt obtained from Rockefeller cultivar wheat grain during the years of research was 77.6%, and the highest value was obtained for wheat malt produced from wheat grain in the second year of research on farm C. Comparing the average extractivity, diastatic power and attenuation of wheat malts obtained from Rockefeller cultivar winter wheat grain collected from field and canopy experiments, it was observed that the extractivity of malts from canopy experiments was at a similar level, diastatic power was 8.45% lower, and attenuation was at the same level in relation to the discussed parameters from field experiments (Table 1, Table 2, Table 3, Table 4 and Table 5).

### 2.6. Cytolytic Modification in Wheat Malts from Commodity Fields Experiment

**Figure 1:**
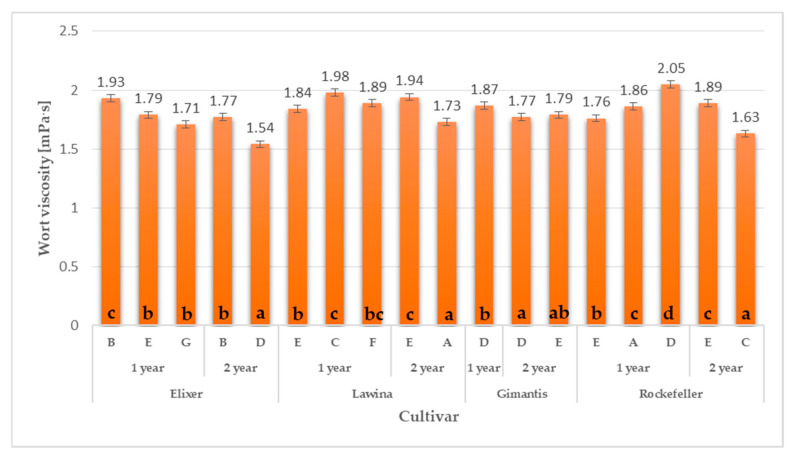
Wort viscosity obtained from winter wheat grain obtained in canopy experiments. Mean values for wort viscorsity with different letters (a–d) are significantly different (*p* < 0.05). The letters A–G indicate the location of the farm where the wheat was grown.

**Figure 2:**
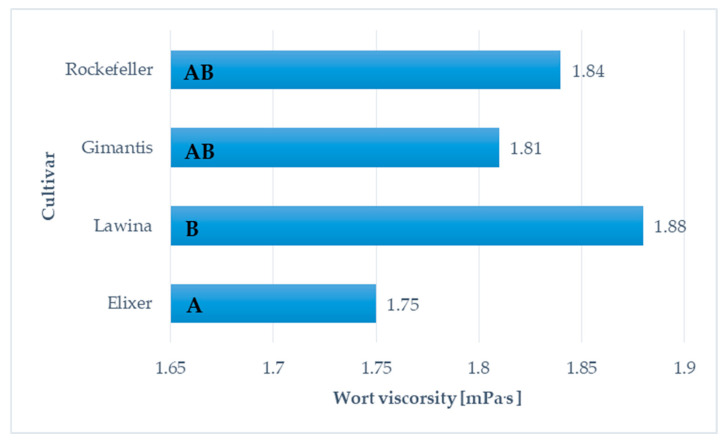
Average wort viscosity obtained from winter wheat grain obtained in canopy experiments Mean values for wort viscorsity with different letters (A-B) are significantly different (*p* < 0.05).

The average viscosity of worts obtained from wheat malt produced from winter wheat grain of the Elixer cultivar collected from field experiments was 1.75 mPa·s, and the lowest value was obtained for the raw material obtained in the second year of research on farm D. Comparing the average viscosity of worts obtained from wheat malt obtained from winter wheat grain of the Elixer cultivar from field and canopy experiments, it was observed that the viscosity of worts from canopy experiments was 7.89% lower in relation to the discussed parameter from field experiments (Figure 1 and Figure 2; Table 1, Table 2, Table 3 and Table 4).

The average viscosity of worts obtained from wheat malt produced from winter wheat grain of the Lawina cultivar collected from field experiments was 1.88 mPa·s, and the lowest value was obtained for the raw material obtained in the second year of research at farm A. Comparing the average viscosity of worts obtained from wheat malt obtained from winter wheat grain of the Lawina cultivar in the analysed years of research from field and canopy experiments, it was observed that the viscosity of wort from canopy experiments was 6.47% lower in relation to the discussed parameter from field experiments (Figure 1 and Figure 2; Table 1, Table 2, Table 3 and Table 4).

The average viscosity of worts obtained from wheat malt produced from winter wheat grain of the Gimantis cultivar collected from field experiments was 1.81 mPa·s, and the lowest value was obtained for the raw material in the second year of research on farm D. Comparing the average viscosity of worts obtained from wheat malt obtained from winter wheat grain of the Gimantis cultivar from field and canopy experiments, it was observed that the viscosity of worts obtained from wheat malt from canopy experiments was 10.40% lower in relation to the discussed parameter from field experiments in the analysed years of research (Figure 1 and Figure 2; Table 1, Table 2, Table 3 and Table 4).

The average viscosity of worts obtained from wheat malt produced from Rockefeller winter wheat grain collected from canopy experiments was 1.84 mPa·s, and the lowest value was obtained for the raw material obtained in the second year of research on farm C. Comparing the average viscosity of worts obtained from wheat malt obtained from Rockefeller winter wheat grain from field and canopy experiments, it was observed that the viscosity of worts obtained from wheat malt produced from Rockefeller winter wheat grain from field experiments was at the same level in relation to the discussed parameter (Figure 1 and Figure 2; Table 1, Table 2, Table 3 and Table 4).

## 3. Discussion

### 3.1. Extractivity in Wheat Malts

Extractivity is one of the most important quality parameters for wheat malts, as it determines how much chemical compounds enter the wort during mashing while affecting the efficiency of the technological process. For wheat malt, the extract content should not be lower than 83.0% [14], the optimum value is a minimum of 85.0% [15] and the influence of the crop year on the value of the parameter in question and, to a lesser extent, the variety of the raw material is important [16]. These authors also showed a high negative correlation between wheat malt extract and nitrogen content in unmalted grain and malt, while positively highly correlated with Kolbach number value and malt crispness. In the analysed research years, after the malting process, the extract content of malt obtained from the grain of the analysed winter wheat varieties was determined.

In a study by Boros et al. [9] involving a search for winter wheat families for wheat malt production, the extractivity of the malts obtained was 83.4% s. m. on average. For wheat malts obtained from Croatian wheat varieties, the extractability was between 82.5 and 83.1% s. m. [17]; from Thai wheat varieties, it was in the range of 83.3–83.9% d.m., while for commercial wheat malts, it ranged from 79.7–83.8% d.m. [18]. The varying endosperm hardness and degree of granulation did not affect the extractability of wheat malts and ranged from 75.17 to 77.40% d.m. [19]. The extract content of malts obtained from different varieties of winter wheat grown in the Czech Republic ranged from 82.3 to 85.4% d.m. [16]. In a study by Belcar et al. [20], five-day wheat malts obtained from wheat grain of the Elixer, Rockefeller and Gimantis varieties had average extractivities of 81.85% d.m., 81.71% d.m. and 87.13% d.m., respectively. In a study by Jin et al. [21], the extractable content of wheat malts was in the range of 79.30–82.30% d.m.; in the evaluation by Depraetere et al. [22], it was in the range of 75.00–86.90% d.m.; and in the study by Hu et al. [23], it was 80.8% s. m. Blšáková et al. [24] determined the extractability of the tested raw material to be 66.7%. In a recent study by Gugino et al. [25], the extractability of wheat malts was between 84.3 and 84.7%.

### 3.2. Diastatic Power in Wheat Malts

The ability to decompose starch to reducing sugars by amylolytic enzymes, mainly β-amylase, i.e., diastatic power, significantly depends on the variety used [16]. The activity of amylolytic enzymes, determined as diastatic power (mainly β-amylase) of the grain and subsequently of the malt, also depends on the sum of precipitation occurring during the growing season. Too low a precipitation sum results in low amylolytic activity of the grain, which is an undesirable phenomenon in malting [26]. The reduced value of the diastatic power parameter (i.e., amylolytic activity) can be increased by the application of gibberellic acid during malting of the grain [16]. According to Psota and Musilová [15], the diastatic power value of wheat malts should not be lower than 250 units W-K (Windisch–Kolbach), and the optimum values are above 350 unit W-K.

According to various literature sources, the diastatic power of the obtained wheat malts ranged from 290 to 470 units W-K [9]. In a study by Psota et al. [16], the diastatic strength of wheat malts obtained from winter wheat grain ranged from 248 to 401 units W-K. In the study by Belcar et al. [20], five-day wheat malts obtained from wheat grain of the Elixer, Rockefeller and Gimantis cultivars had average diastatic strengths of 331 units W-K, 305 units W-K and 315 units W-K, respectively. Jin et al. [21] obtained wheat malts characterised by diastatic strengths ranging from 384 to 496 units W-K. In the study by Gugino et al. [25], the diastatic power of wheat malts was in the range of 374.51–375.59 W-K.

### 3.3. Degree of Final Attenuation of Wheat Malts

For the wheat grain cultivars used, the malting and malt-mashing process significantly affect the fermentation of the wort [16]. A minimum value of 83.0% is considered to be the optimum attenuation value for wheat wort, while a value below 80.0% is unacceptable [15].

In studies by other authors, the degree of attenuation of wheat worts was in the range of 78.7–93.3% [19] and averaged 82.9% [9]. Wheat malts obtained from winter wheat cultivars grown in the Czech Republic were characterised by a degree of attenuation of 78.6–81.4% [16]. In a study by Belcar et al. [20], five-day wheat malts obtained from wheat grain of the Elixer, Rockefeller and Gimantis cultivars were characterised by an average attenuation of 82.4%, 80.1% and 80.9%, respectively. In a study by Gugino et al. [25], the attenuation of wheat malts was between 81.7 and 81.9%.

### 3.4. Viscosity of Worts

The lack of husk in the wheat grain and the high arabinoxylan content in the chemical composition not only affects the filtration time but also increases the viscosity of the obtained wheat worts [15,16,18,27,28]. This parameter depends significantly on the wheat cultivar used [15,16]. The value of the wort viscosity parameter can be used as an indicator of the filtration process, and the lower its value, the lower the resistance of the liquid during flow [24]. According to Titze et al. [14], wheat worts characterised by viscosities above 1.80 mPa·s are unacceptable to brewers, while according to authors Psota and Musilová [15], this value should be up to 2.00 mPa·s.

In studies by other authors, the average viscosity of wheat worts was 1.79 mPa·s [9] and ranged from 1.687 to 2.314 mPa·s [16]. The viscosity of worts obtained from Croatian wheat cultivars was at an average level of 1.54 mPa·s [17], for varying endosperm hardness and granulation, it was at a similar level (1.425 mPa·s; [19]). In a study by Belcar et al. [20], five-day wheat malts obtained from wheat grains of the Elixer, Rockefeller and Gimantis cultivars had average viscosities of 2.47 mPa·s, 2.41 mPa·s and 1.80 mPa·s, respectively. In a study by Zhuang et al. [29], the average viscosity of the wort obtained from 100% unmalted wheat grain was 1.79 mPa·s.

## 4. Materials and Methods

### 4.1. Materials

The research material consisted of grain of four winter wheat cultivars: Elixer, Lawina, Gimantis and Rockefeller (class C wheat, fodder cultivars) from field experiments conducted in the 2020/2021, 2021/2022 and 2022/2023 growing seasons in Jelcz-Laskowice (51°21’ N; 17°35’ W) belonging to IUNG-PIB, Department of Herbology and Cultivation Techniques in Wrocław (Poland). The research also used grain of the same cultivars grown on seven commercial farms in canopy experiments in the 2020/2021 and 2021/2022 growing seasons in Łańcut and Przeworsk districts, Podkarpackie province. The conditions for conducting field and canopy experiments and the method of preparing malts were described in Belcar and Gorzelany [30]. In short, representative samples of raw materials were cleaned on Vögel sieves, and the grain fraction >2.5 mm was obtained for testing, spread on metal plates for germination and soaked until the grain moisture content reached 45% (sprayed twice with tap water from an atomiser at 15 ± 1 °C during the day at 12 h intervals). The sample dishes were placed in a climate cabinet with a relative humidity of 90% and a temperature of 15 ± 1 °C. After 5 days of germination, the raw material was dried in a laboratory dryer with the following operating times and then germinated: 15 h at 40 ◦C, 3 h at 50 °C, 3 h at 65 °C and 2 h at 80 °C.

### 4.2. Qualitative Analysis of the Malts Obtained

The extract content of the obtained malts was determined according to the 4.5.1 EBC (European Brewery Convention) method [31], diastatic power according to the 4.12.1 EBC method [32], final attenuation degree according to the 4.11.1 EBC method [33] and laboratory wort viscosity according to the 4.6.1 EBC method [34]. All analyses were performed in triplicate.

### 4.3. Statistical Analysis

All the analyses described were performed in three parallel replications. The results were developed using the statistical program Statistica 13.3. The interpretation of the results was performed using one-way ANOVA (Tukey’s test) at a significance level of α = 0.05 for the individual malt traits determined depending on the wheat cultivar. The study of the effect of wheat variety and nitrogen fertilisation level on the degree of hydrolytic and cytolytic modification of wheat malts was performed using multi-way analysis of variance (HSD-Tukey) at a significance level of α = 0.05.

## 5. Conclusions

The malting process influenced the degree of hydrolytic and cytolytic modification of malts obtained from selected winter wheat cultivars grown in different nitrogen fertilisation variants in field experiments. Nitrogen fertilisation at a dose of 60 kg N·ha^−1^ had a positive effect on the extractive value of wheat malts. The value of diastatic power in the obtained malts varied depending on the variety and the level of nitrogen fertilisation, and the best results were obtained for the Elixer and Rockefeller cultivars at the applied fertilisation levels of 60 and 80 kg N·ha^−1^. The fermentation of the wort obtained from the analysed wheat malts at the applied nitrogen fertilisation doses was at a similar level, except for the malt obtained from wheat grain fertilised with a nitrogen dose of 40 kg N·ha^−1^, for which significantly lower values were obtained. The viscosity parameter of wheat malts defining the degree of cytolytic modification varied depending on the cultivar used and the level of nitrogen fertilisation, but was generally characterised by an increased value. In the canopy experiments, with nitrogen fertilisation at the level of 60 kg N·ha^−1^, the Elixer cultivar had the best indicators of the degree of hydrolytic and cytolytic modification. The obtained results are of great importance in the brewing industry due to the efficiency of the mashing process and the share of sugars in the wort, which is associated with a higher share of ethyl alcohol, which is a very desirable phenomenon in brewing. In addition, the lower viscosity of the obtained wort reduces the friction caused by the solution during flow through the technological pipes, and thus reduces industrial damage.

## Figures and Tables

**Table 1:** Extractivity (% d.m.) of wheat malts obtained from wheat grain from field experiments in the study years analysed.

Growing Season	Cultivar	Control	Variant I	Variant II	Variant III
2020/21	Elixer	77.01C^b^ ± 0.25	71.55A^a^ ± 0.44	80.39A^b^ ± 0.36	68.51A^a^ ± 0.28
	Lawina	82.59D^a^ ± 0.18	86.12C^b^ ± 0.21	88.03C^b^ ± 0.25	88.08D^b^ ± 0.20
	Gimantis	81.39D^a^ ± 0.39	84.96BC^bc^ ± 0.27	85.67BC^c^ ± 0.25	83.71C^ab^ ± 0.27
	Rockefeller	77.60C^a^ ± 0.18	87.89C^c^ ± 0.17	86.75C^c^ ± 0.23	84.78C^b^ ± 0. 20
2021/22	Elixer	66.67A^a^ ± 0.16	77.32B^b^ ± 0.32	80.14A^c^ ± 0.34	79.56B^b^ ± 0.38
	Lawina	78.28C^a^ ± 0.25	76.91B^a^ ± 0.22	79.09A^a^ ± 0.27	78.61B^a^ ± 0.34
	Gimantis	82.07D^a^ ± 0.18	86.16C^b^ ± 0.24	86.95C^b^ ± 0.18	83.31C^ab^ ± 0.23
	Rockefeller	72.02B^a^ ± 0.18	84.34BC^b^ ± 0.26	83.17AB^b^ ± 0.22	82.74BC^b^ ± 0.29
2022/23	Elixer	88.21E^b^ ± 0.35	80.72B^a^ ± 0.26	85.66BC^b^ ± 0.13	85.51CD^b^ ± 0.27
	Lawina	83.67D^a^ ± 0.24	88.68C^b^ ± 0.13	89.3C^b^ ± 0.16	85.57CD^a^ ± 0.22
	Gimantis	78.85C^a^ ± 0.15	85.97C^b^ ± 0.15	88.95C^b^ ± 0.21	80.74B^a^ ± 0.25
	Rockefeller	87.70E^b^ ± 0.10	85.85C^b^ ± 0.15	80.02A^a^ ± 0.15	87.80D^b^ ± 0.09
Average for the years		2020/21		82.19^1,2^ ± 5.88	
		2021/22		79.83^1^ ± 5.17	
		2022/23		85.20^2^ ± 3.43	
Average for the cultivation		Elixer		78.44^A^ ± 6.73	
		Lawina		83.75^B^ ± 4.55	
		Gimantis		84.06^B^ ± 2.92	
		Rockefeller		83.39^B^ ± 4.80	
Average for the fertilisation		Control		79.67^a^ ± 6.12	
		Variant I		83.04^ab^ ± 5.26	
		Variant II		84.51^b^ ± 3.77	
		Variant III		82.41^ab^ ± 5.29	
Average				82.41 ± 2.19	

Data are expressed as a mean values (n = 3) ± SD; SD—standard deviation. Mean values within rows with different letters are significantly different (A–E statistical differences between cultivars; and a–c statistical differences at the analysed levels of nitrogen fertilisation, and 1–2 statistical differences at the growing season, with *p* < 0.05).

**Table 2:** Diastatic power (unit W-K) of wheat malts obtained from wheat grain from field experiments in the study years analysed.

Growing Season	Cultivar	Control	Variant I	Variant II	Variant III
2020/21	Elixer	475F^a^ ± 6	466F^a^ ± 1	476E^a^ ± 5	489E^a^ ± 5
	Lawina	357C^a^ ± 0	365D^a^ ± 5	359C^a^ ± 7	358BC^a^ ± 8
	Gimantis	387D^a^ ± 7	407E^b^ ± 0	390D^ab^ ± 5	386C^a^ ± 4
	Rockefeller	469F^b^ ± 1	427E^a^ ± 3	483E^b^ ± 9	477DE^b^ ± 10
2021/22	Elixer	366C^a^ ± 18	353CD^a^ ± 2	354C^a^ ± 4	355BC^a^ ± 3
	Lawina	369C^a^ ± 0	374D^a^ ± 4	372CD^a^ ± 2	372C^a^ ± 9
	Gimantis	317B^b^ ± 6	306B^a^ ± 10	308A^a^ ± 4	305A^a^ ± 14
	Rockefeller	444E^a^ ± 2	452F^a^ ± 2	451E^a^ ± 5	464D^a^ ± 8
2022/23	Elixer	332B^a^ ± 4	336C^a^ ± 4	338B^a^ ± 2	331B^a^ ± 5
	Lawina	256A^a^ ± 0	267A^a^ ± 2	318A^c^ ± 10	302A^b^ ± 6
	Gimantis	260A^a^ ± 9	270A^a^ ± 4	323AB^b^ ± 4	353BC^c^ ± 7
	Rockefeller	248A^a^ ± 4	319BC^b^ ± 11	365C^c^ ± 4	369C^c^ ± 0
Average for the years		2020/21		423^3^ ± 52	
		2021/22		373^2^ ± 54	
		2022/23		312^1^ ± 40	
Average for the cultivation		Elixer		389^B^ ± 65	
		Lawina		339^A^ ± 43	
		Gimantis		334^A^ ± 49	
		Rockefeller		414^C^ ± 73	
Average for the fertilisation		Control		357^a^ ± 79	
		Variant I		362^ab^ ± 66	
		Variant II		378^b^ ± 61	
		Variant III		380^b^ ± 64	
Average				369 ± 33	

Data are expressed as a mean values (n = 3) ± SD; SD—standard deviation. Mean values within rows with different letters are significantly different (A–F, statistical differences between cultivars; and a–c, statistical differences at the analysed levels of nitrogen fertilisation, and 1–3 statistical differences at the growing season, with *p* < 0.05).

**Table 3:** Degree of final attenuation (%) of wheat malts obtained from wheat grain from field experiments in the study years analysed.

Growing Season	Cultivar	Control	Variant I	Variant II	Variant III
2020/21	Elixer	74.8B^c^ ± 0.5	59.6B^a^ ± 0.4	64.9A^b^ ± 0.6	62.2A^b^ ± 0.1
	Lawina	68.1A^b^ ± 0.4	56.3A^a^ ± 0.3	65.5A^b^ ± 0.3	75.7C^c^ ± 0.7
	Gimantis	80.2D^b^ ± 0.6	74.4D^a^ ± 0.4	76.2B^ab^ ± 0.1	77.7CD^ab^ ± 0.2
	Rockefeller	80.1D^b^ ± 0.1	72.5C^a^ ± 0.2	75.3B^a^ ± 0.3	80.4D^b^ ± 0.3
2021/22	Elixer	80.0CD^a^ ± 0.4	82.3F^ab^ ± 0.3	88.3D^c^ ± 0.5	85.5E^bc^ ± 0.6
	Lawina	77.7C^ab^ ± 0.3	80.1E^ab^ ± 0.5	81.7C^b^ ± 0.0	76.6C^a^ ± 0.4
	Gimantis	79.3C^a^ ± 0.6	83.1F^ab^ ± 0.1	85.5D^b^ ± 0.4	84.4E^b^ ± 0.2
	Rockefeller	82.8D^b^ ± 0.0	72.2C^a^ ± 0.1	80.5C^b^ ± 0.3	81.1D^b^ ± 0.3
2022/23	Elixer	77.6C^b^ ± 0.4	70.9C^a^ ± 0.2	85.2D^c^ ± 0.0	82.9DE^c^ ± 0.5
	Lawina	83.1D^c^ ± 0.1	79.8E^bc^ ± 0.0	75.5B^b^ ± 0.5	70.3B^a^ ± 0.2
	Gimantis	78.8C^b^ ± 0.3	80.6EF^b^ ± 0.3	77.4B^ab^ ± 0.4	80.0D^b^ ± 0.1
	Rockefeller	76.1BC^a^ ± 0.6	77.9DE^ab^ ± 0.4	81.2C^b^ ± 0.5	80.5D^b^ ± 0.4
Average for the years		2020/21		71.5^1^ ± 7.7	
		2021/22		81.3^2^ ± 3.9	
		2022/23		78.6^2^ ± 4.1	
Average for the cultivation		Elixer		76.2^AB^ ± 9.7	
		Lawina		74.2^A^ ± 7.8	
		Gimantis		79.8^B^ ± 3.3	
		Rockefeller		78.4^B^ ± 3.5	
Average for the fertilisation		Control		78.2^b^ ± 4.0	
		Variant I		74.1^a^ ± 8.6	
		Variant II		78.1^b^ ± 7.3	
		Variant III		78.1^b^ ± 6.5	
Average				77.1 ± 2.9	

Data are expressed as a mean values (n = 3) ± SD; SD—standard deviation. Mean values within rows with different letters are significantly different (A–F, statistical differences between cultivars; and a–c, statistical differences at the analysed levels of nitrogen fertilisation, and 1–2 statistical differences at the growing season, with *p* < 0.05).

**Table 4:** Viscosity (mPa·s) of worts obtained from wheat malts obtained from wheat grain from field experiments during the study years analysed.

Growing Season	Cultivar	Control	Variant I	Variant II	Variant III
2020/21	Elixer	1.85C^c^ ± 0.07	1.73AB^b^ ± 0.07	1.55A^a^ ± 0.05	1.96B^d^ ± 0.10
	Lawina	1.83BC^b^ ± 0.21	1.66A^a^ ± 0.23	1.82B^b^ ± 0.17	2.00BC^c^ ± 0.11
	Gimantis	2.29E^c^ ± 0.19	2.07D^b^ ± 0.11	2.25DE^c^ ± 0.05	1.75A^a^ ± 0.08
	Rockefeller	2.24E^c^ ± 0.20	1.75B^a^ ± 0.08	2.26DE^c^ ± 0.10	1.91B^b^ ± 0.09
2021/22	Elixer	1.40A^a^ ± 0.10	1.98CD^b^ ± 0.14	2.09C^c^ ± 0.08	2.11CD^c^ ± 0.04
	Lawina	1.98D^a^ ± 0.14	1.97CD^a^ ± 0.14	2.15CD^b^ ± 0.15	2.18D^b^ ± 0.16
	Gimantis	2.17E^a^ ± 0.15	2.10D^a^ ± 0.10	2.31E^b^ ± 0.13	2.10CD^a^ ± 0.05
	Rockefeller	1.37A^a^ ± 0.18	1.91C^c^ ± 0.13	1.89B^c^ ± 0.13	1.73A^b^ ± 0.24
2022/23	Elixer	1.87C^a^ ± 0.14	2.21D^c^ ± 0.29	2.06C^b^ ± 0.12	2.00BC^b^ ± 0.10
	Lawina	1.76BC^a^ ± 0.17	2.11D^b^ ± 0.11	2.68F^c^ ± 0.08	2.03BC^b^ ± 0.13
	Gimantis	1.44A^a^ ± 0.12	1.67A^b^ ± 0.12	1.94B^c^ ± 0.06	2.09CD^d^ ± 0.09
	Rockefeller	1.74B^b^ ± 0.19	1.97CD^c^ ± 0.12	1.63A^a^ ± 0.24	1.75A^b^ ± 0.09
Average for the years		2020/21		1.93^1^ ± 0.23	
		2021/22		1.97^1^ ± 0.26	
		2022/23		1.93^1^ ± 0.29	
Average for the cultivation		Elixer		1.90^A^ ± 0.24	
		Lawina		2.01^B^ ± 0.26	
		Gimantis		2.02^B^ ± 0.27	
		Rockefeller		1.85^A^ ± 0.25	
Average for the fertilisation		Control		1.83^a^ ± 0.32	
		Variant I		1.93^b^ ± 0.19	
		Variant II		2.05^c^ ± 0.31	
		Variant III		1.97^b^ ± 0.15	
Average				1.94 ± 0.07	

Data are expressed as a mean values (n = 3) ± SD; SD—standard deviation. Mean values within rows with different letters are significantly different (A–F, statistical differences between cultivars; and a–d, statistical differences at the analysed levels of nitrogen fertilisation, and 1 statistical differences at the growing season, with *p* < 0.05).

**Table 5:** Extractivity, diastatic power and degree of attenuation of wheat malts obtained from winter wheat grain obtained in canopy experiments.

Farm	Cultivar	Year of Research	Extractivity (% d.m.)	Diastatic Power (Units W-K)	Degree of Attenuation (%)
B	Elixer	I	78.14^a^ ± 0.36	389^b^ ± 12	76.6^ab^ ± 0.4
B		II	75.50^a^ ± 0.60	346^a^ ± 7	80.4^b^ ± 0.3
E		I	82.53^b^ ± 0.47	371^b^ ± 23	78.2^ab^ ± 0.5
G		I	82.74^b^ ± 0.53	348^a^ ± 14	80.1^b^ ± 0.1
D		II	75.49^a^ ± 0.34	371^b^ ± 13	75.1^a^ ± 0.6
Average			78.88 ± 3.59	365 ± 18	78.1 ± 2.3
E	Lawina	I	88.67^d^ ± 0.33	323^b^ ± 9	80.0^d^ ± 0.3
E		II	83.41^c^ ± 0.65	308^ab^ ± 10	78.1^cd^ ± 0.5
C		I	78.82^ab^ ± 0.45	301^a^ ± 11	70.3^a^ ± 0.7
F		I	77.33^a^ ± 0.68	321^b^ ± 14	73.4^ab^ ± 0.2
A		II	82.22^bc^ ± 0.78	318^b^ ± 5	75.5^bc^ ± 0.4
Average			82.09 ± 4.43	314 ± 9	75.5 ± 4.4
D	Gimantis	I	87.84^b^ ± 0.16	305^a^ ± 6	65.7^a^ ± 0.3
D		II	82.14^a^ ± 0.44	300^a^ ± 5	80.2^b^ ± 0.4
E		II	78.75^a^ ± 0.38	302^a^ ± 3	82.5^b^ ± 0.1
Average			82.91 ± 4.59	302 ± 3	76.1 ± 9.1
E	Rockefeller	I	83.11^b^ ± 0.62	367^a^ ± 12	78.3^b^ ± 0.4
E		II	85.66^b^ ± 0.35	367^a^ ± 10	75.1^b^ ± 0.2
A		I	86.06^b^ ± 0.60	387^b^ ± 11	78.7^b^ ± 0.3
D		I	82.23^b^ ± 0.30	394^b^ ± 8	70.3^a^ ± 0.1
C		II	73.34^a^ ± 0.67	380^ab^ ± 11	85.5^c^ ± 0.5
Average			82.08 ± 5.15	379 ± 12	77.6 ± 5.6

Data are expressed as a mean values (n = 3) ± SD; SD—standard deviation. Mean values within rows for quality indicators with different letters (a–d) are significantly different (*p* < 0.05).

## Data Availability

Samples of the compounds used in the research are available from the authors.
